# Discovery and verification of mmu_Circ_26986/hsa_Circ_0072463 as a potential biomarker and intervention target for sepsis-associated acute kidney injury

**DOI:** 10.1007/s00018-023-05079-x

**Published:** 2024-03-28

**Authors:** Xujun Peng, Huiling Li, Wenbo Zhang, Dongshan Zhang

**Affiliations:** 1 Department of Emergency Medicine, Second Xiangya Hospital, Central South University, Changsha, Hunan People’s Republic of China; 2 Emergency Medicine and Difficult Diseases Institute, Second Xiangya Hospital, Central South University, Changsha, 410011 Hunan People’s Republic of China; 3 Department of Ophthalmology, Second Xiangya Hospital, Central South University, Changsha, Hunan People’s Republic of China; 4 Department of Nephrology, Second Xiangya Hospital, Central South University, Changsha, Hunan People’s Republic of China; 5 Boya College of Macau University of Science and Technology, Taipa, China

**Keywords:** mmu_Circ_26986, hsa_Circ_0072463, Biomaker, AKI, SA-AKI, Apoptosis, LPS, CLP, miRNA-29b-1-5p, PAK7

## Abstract

Approximately 60% of septic patients developed acute kidney injury (AKI). The mortality rate of septic AKI (SA-AKI) is two to three times higher than that of septic without AKI (SA-non-AKI). The actual functions and mechanisms of CircRNAs in the pathophysiology of SA-AKI remain incompletely understood. Herein, we observed that the mmu_Circ_26986 could be induced by lipopolysaccharide (LPS) and cecum ligation and puncture (CLP) in BUMPT cell line and C57BL/6 mouse kidney, respectively. Functionally, mmu_Circ_26986 suppressed BUMPT cell apoptosis induced by LPS. Mechanistically, mmu_Circ_26986 sponged miRNA-29b-1-5p to upregulate the expression of PAK7. Overexpression of mmu_Circ_26986 ameliorated the progression of CLP-stimulated AKI through miRNA-29b-1-5p/PAK7 axis. In addition, we found that hsa_Circ_0072463, homologous to mmu_Circ_26986, suppressed LPS-induced HK-2 cells apoptosis via regulation of miRNA-29b-1-5p/PAK7 axis. Furthermore, sepsis patients with AKI had a higher level of hsa_Circ_0072463 compared to those without AKI. The sensitivity, specificity and AUC of hsa_Circ_0072463 were 78.8%, 87.9% and 0.866, respectively. Spearman's test indicated a noticeable positive correlation between plasma hsa_Circ_0072463 and serum creatinine in sepsis patients (r = 0.725). In summary, this study reveals that the mmu_Circ_26986/hsa_Circ_0072463 miRNA-29b-1-5p/PAK7 axis mediates septic AKI, and hsa_Circ_0072463 is a potential diagnostic marker for septic AKI.

**Supplementary Information:**

The online version contains supplementary material available at 10.1007/s00018-023-05079-x.

## Introduction

Approximately 60% of patients with sepsis suffer from sepsis-associated acute kidney injury (SA-AKI), exacerbating sepsis morbidity and mortality [1]. Therefore, it is crucial to early identify SA-AKI and investigate its underlying mechanism [2]. Recent data have identified more than ten proteins, such as TIMP2 and IGFBP7, with potential diagnosis values for SA-AKI [3,4]. Additionally, three underlying mechanisms, including microcirculatory dysfunction [5], inflammation [6], and metabolic reprogramming [7], contribute to the progression of SA-AKI. However, there is currently no effective treatment method other than kidney replacement therapy to prevent the progression of SA-AKI [8–10]. Hence, our goal is not only to explore the pathophysiology of SA-AKI, but also to identify better early biomarkers for it. The significant aspect is that the new molecule serves as both an intervention target and a potential biomarker for SA-AKI.

Circular RNAs (CircRNAs), a specific class of RNA molecules, belong to non-coding RNAs (ncRNAs) [11]. In comparison with linear RNAs, CircRNAs exhibit greater stability, possess a longer lifespan, and demonstrate resistance to RNase R, making them ideal biomarkers for human diseases [12,13]. Moreover, CircRNAs are responsible for the progression of various diseases and have become therapeutic targets [14]. Recent research has identified several CircRNAs responsible for the progression of SA-AKI [15]. Only one study has indicated that Circ_0020339 promotes the development of SA-AKI and acts as a diagnostic marker [16]. In this study, we focus on mmu_Circ_26986, located in Arl15, whose role and diagnostic value in SA-AKI remain largely unclear.

Our study indicated that lipopolysaccharide (LPS) and cecum ligation and puncture (CLP) could induce the expression of mmu_Circ_26986 both in cell and animal experiments. Mmu_Circ_26986 suppressed LPS-stimulated apoptosis in BUMPT cells by targeting the miRNA-29b-1-5p/PAK7 axis. CLP-induced SA-AKI in mice was ameliorated by the overexpression of mmu_Circ_26986. Finally, we found that hsa_Circ_0072463, homologous to mmu_Circ_26986, serves as an early diagnostic marker for SA-AKI.

## Materials and methods

### Antibodies and reagents

Anti-PAK7 and anti-β-tubulin antibodies were procured from Proteintech (USA). Anti-C3 (9662) and CC3 (9664) antibodies were supplied by Cell Signaling Technology (USA). Secondary antibodies were obtained from Affinity (USA). FITC-Annexin-V-Apoptosis-Detection-Kit-I (556,547; BD Pharmingen, USA); luciferase-assay-kit (BioVision, USA); AG-SYBR-Green-Pro-Taqhs-premix (Accurate Biotechnology, China); LPS (L2880; Sigma, USA).

### Cell culture and treatment

BUMPT and HK-2 cell lines were cultured with DMEM (Sigma-Aldrich) containing 10% FBS and 1% penicillin–streptomycin at 37 °C and 5% CO2, and then treated with LPS for 24 h at 300 mg/mL or 50 mg/mL, respectively. Cells were transfected with mmu_Circ_26986 siRNA (100 nM) or plasmid, hsa_Circ_0072463 plasmid (1 ug/ml), miRNA-29b-1-5p mimic (100 nM), miRNA-29b-1-5p inhibitor (100 nM), PAK7 siRNA, or negative control (Ruibo, China) using Lipofectamine-2000 (Life Technologies, USA).

### Animal models

Circ_26986 or control expression vectors (25 μg DNA/injection) were administered through the tail vein of C57BL/6 J male mice (8–10 weeks old), and then subjected to CLP, saline injection as a sham operation as a control, respectively. Kidney tissues were acquired for assessing renal morphology and function at 18 h after CLP treatment. The study involved animal testing in adherence to the guidelines set by the Institutional Committee for the Care and Use of Laboratory Animals at the Second Xiangya Hospital. Throughout the experiments, the animals were provided unrestricted access to standard water and food and followed a 12-h light–dark cycle.

### Cecum ligation and puncture model

C57 mice weighing 21–25 g were selected and fasted for 12 h before surgery. After anaesthesia was administered by intraperitoneal injection according to body weight, the animals were fixed supine on a surgical plate, the abdominal surgical regions were routinely disinfected and dehairing was performed, and a 2-cm-long incision was made on the abdominal wall with a scalpel in aseptic conditions. An 18-gauge needle was used to perforate the ligated end and a small amount of faeces was squeezed out, avoiding damage to the blood vessels as much as possible, and then the skin and peritoneum were closed intermittently with a 4-gauge silk suture, while 50 ml/kg saline was injected for antishock.

### qRT-PCR analysis

Trizol Reagent (Invitrogen) was utilized for total RNA extraction from BUMPT cells, HK-2 cells, and kidneys of C57BL/6 J mice. Next, approximately 40 ng of total RNA was reverse transcribed to cDNA using the Prime-Script-RT-Reagent-Kit and the gDNA-Eraser-Kit (RR047A; TaKaRa, Japan). qRT-PCR and SYBR Green (AG11728; Accurate Biotechnology (HUNAN) CO., LTD, ChangSha China) were employed to detect the expression levels of circRNA, micoRNA, and target gene mRNA. Relative quantification was performed by Roche LC 480 determination of ΔCt values (F. Hoffmann-La Roche, Ltd.).

Primers:

Circ_26986 (F: GAACGAACTGCACTCCGCTCTC, R: GCTGCTGGCTTGTCTTGATGATTG)

miRNA-29b-1-5P (F: GCACCGTGCTGGTTTCATATGG, R: ATCCAGTGCAGGGTCCGAGG

RT primer: ATCCAGTGCAGGGTCCGAGG)

hsa_Circ_0072463 (F: TTCCGATGACCAGTTACACAA, R: TTGGTAGTAGCGGCTCCAGT)

PAK7 (F: CTGGGAGAGGTTTGGGAGGAGAG, R: AGGGAACTACTACGGCTGGGAAG)

U6 (F: AGAGAAGATTAGCATGGCCCCTG, R: CAGTGCAGGGTCCGAGGT)

Endogenous Reference Human (F: CCTGGCACCCAGCACAAT, R: GGGCCGGACTCGTCATA)

Endogenous Reference Mouse (F: GGCTGTATTCCCCTCCATCG, R: CCAGTTGGTAACAATGCCATGT)

### FISH analysis

BUMPT cell line or kidney tissues were fixed with paraformaldehyde for 5 min and then permeabilized with prehybridization solution and hybridization solution. Mmu_Circ_26986 fluorescent probe and miRNA-29b-1-5p fluorescent probe (Ruibo, China) were hybridized overnight at 37°. On the next day, nuclei were blocked and stained with DAPI, followed by fluorescence imaging analysis using a laser scanning confocal microscope. U6 nuclear positivity and 18S rRNA cytoplasmic positivity were used for references.

### Immunoblot analysis

Briefly, about 30 ng proteins were separated through SDS-PAGE followed by the transfer using 0.22 nm PVDF membrane (Amersham, UK). The membranes were exposed to primary antibody pak7 (1:1000 dilutiofoon), C3 (1:2000 dilution), CC3 (1:1000 dilution), and β-tubulin (1:2000 dilution) for 4°overnight, and then exposed to secondary antibody for 1 h. After PBST washing, the membranes were developed with ECL reagent. β-tubulin is an internal control.

### Flow cytometry assessment

Annexin V-FITC/PI staining was performed to determine apoptosis. Briefly, BUMPT or HK-2 cell lines were harvested and resuspended with binding buffer, and subsequently stained with annexin V-FITC and PI. After incubation for 15 min in the dark, 200 uL of binding buffer was added and examined using a Northern Light flow meter (Cytek Biosciences). Apoptosis rate was the percentage of late apoptotic (AnnexinV + /PI +) and early apoptotic (AnnexinV + /PI-) cells to cells.

### Luciferase reporter assays

The DLR of WT-Luc-PAK7, WT-Luc-Circ_26986, MUT1-Luc-PAK7, MUT2-Luc-PAK7 and MUT-Circ_26986 plasmids were established by Tsingke Biotechnology (Beijing, China), and then co-transfected with miRNA-29–1-5p mimics into BUMPT cell lines for 48 h. Luciferase activity was examined using SpectraMax M5 (Molecular Devices, USA) following normalization with pGMLR-TK as an internal reference.

### Renal function, morphology, and TUNEL staining

The concentrations of BUN and creatinine were detected (Nanjing Jianjian Bioengineering Research Institute, China). H&E staining was used to evaluate the degree of renal tubular injury: renal tubules were markedly dilated and cellular flattening was scored as 1 point; brush border injury was scored as 1 point, detachment was scored as 2 points; titularity was scored as 2 points; and the presence of detached, necrotic cells in the lumen of renal tubules (which did not become tubular or cellular debris) counted as 1 point. TUNEL staining was applied to determine renal cell apoptosis, positively stained cells were counted and stained samples were evaluated using Zeiss microscope-equipped software.

### Human samples

The definition and grading of SA-AKI were referred to the consensus guidelines and KDIGO criteria, respectively. Human blood was collected from healthy volunteers (n = 33), SA-non-AKI patients (n = 33), and SA-AKI patients (n = 33), and then centrifuged to extract plasma or serum, followed by storage at -80°. The research methodology received approval from the Second Review Committee of Xiangya Hospital, People's Republic of China. Prior to participation, all individuals provided informed consent for their involvement in the study.

### Statistical analysis

GraphPad Prism 9.4 was utilized for statistical analyses. All values are shown as mean ± SD. Differences between two or more groups were examined with Student's T-test or one-way ANOVA followed by Tukey's post-hoc test. ROC curve analysis was also performed using GraphPad. The relationship between Circ and creatinine was analyzed by Spearman's correlation coefficients.

## Results

### Mmu_Circ_26986 expression is elevated in LPS-induced BUMPT cells and CLP-induced mice AKI

**Fig. 1:**
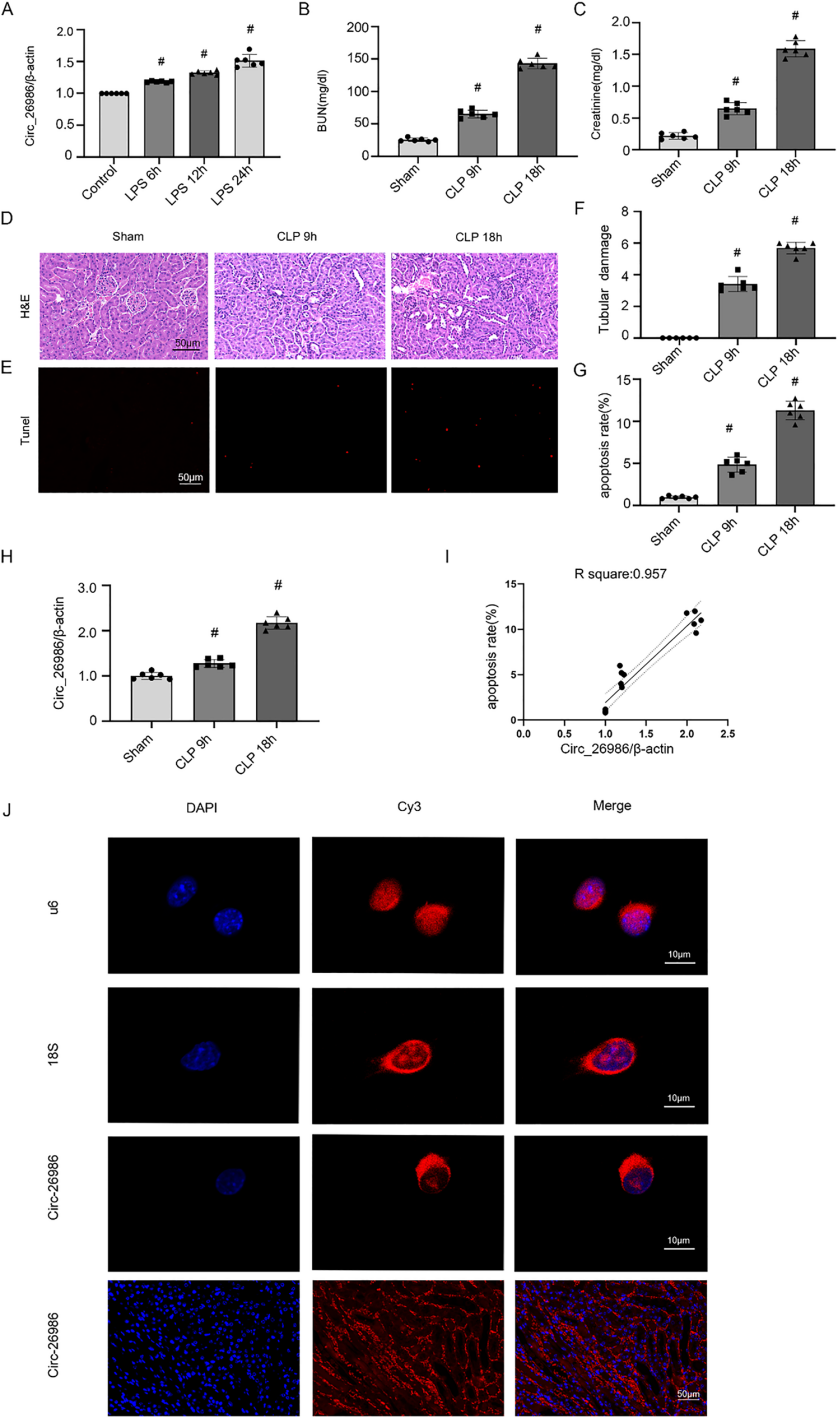
Effect of LPS on Circ_26986 expression in BUMPT cell and CLP mouse models. BUMPT cell line was exposed to LPS (300 μg/mL) for 6, 12 or 24 h. **A** qRT-PCR detection of Circ_26986 expression in cells. C56BL/6 mice were exposed to CLP for 9 and 18 h. Time-dependent increases of BUN (**B**) and serum creatinine (**C**) in CLP-induced sepsis mice. **D**, **E** H&E and TUNEL staining for assessing the degree of impairment. Score bar: 50 µm. **F** H&E damage score. **G** TUNEL positive cells/mm^2^. **H** qRT-PCR was applied to determine Circ_26986 expression. **I** Correlation coefficients of Circ_26986 expression in kidney tissues with CLP-stimulated apoptosis. **J** RNA-FISH assay of Circ_26986 localization in BUMPT cell line and mouse kidney tissues. Nuclear and cytoplasmic labeling were performed with U6 and 18S as the controls, respectively. Finally, Circ_26986 was also localized in the kidney via RNA-FISH assay. Score bar: 10 µm. #p < .05, LPS 12 and 24 h or CLP 9 h and 18 h groups compared with Control or Sham groups

We examined the expression of mmu_Circ_26986 in BUMPT cell line and C57BL/6 mouse model induced by LPS and CLP, respectively. Firstly, qRT-PCR data unveiled that mmu_Circ_26986 was highly expressed at 6 h, gradually increased at 12 h, and peaked at 24 h (Fig. 1A). Secondly, to determine the in vivo expression of mmu_Circ_26986, a sepsis animal model was constructed by CLP in C57BL/6 J mice. Renal function detection indicated a slight increase in BUN and Creatinine induced by CLP at 6 h, reaching a peak at 18 h (Fig. 1B, C). H&E and TUNEL staining verified that CLP gradually exacerbated renal tubular injury and apoptosis at 6 h and 18 h (Fig. 1D-G). The qRT-PCR data also showed that mmu_Circ_26986 was highly expressed at 9 h and peaked at 18 h (Fig. 1H). Correlation analysis revealed a high correlation between the mmu_Circ_26986-fold change (R = 0.957) and the renal cell apoptosis rate (Fig. 1I). FISH analysis demonstrated that mmu_Circ_26986 mainly localized in the cytoplasm of BUMPT and mouse kidney tubular cell lines (Fig. 1J). These findings imply that mmu_Circ_26986 is related to the progression of SA-AKI.

### Downregulation of mmu_Circ_26986 enhances LPS-stimulated BUMPT cell apoptosis

**Fig. 2:**
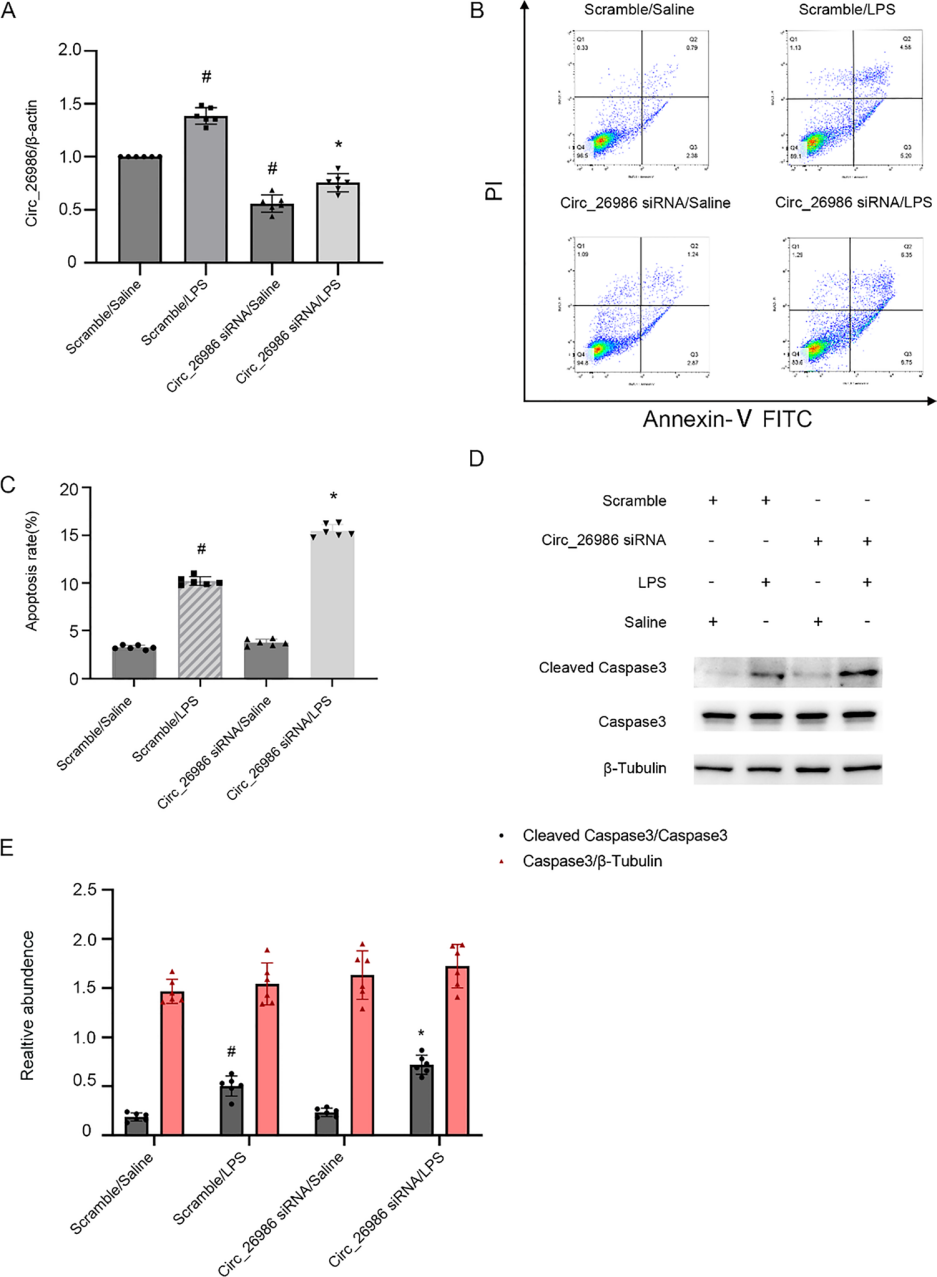
siRNA of Circ_26986 enhances LPS-stimulated apoptosis in BUMPT cells. Following transfection with Circ_26986 siRNA (100 nM) or scramble (SC), the cell line was exposed to LPS for 24 h. **A** qRT-PCR was utilized to determine Circ_26986 levels. **B** Flow cytometry was used to examine BUMPT cell apoptosis. The cells in Q4, Q3, Q2 and Q1 regions represent normal cells, early apoptotic cells, late apoptotic cells and necrotic cells, respectively. **C** Representative apoptotic rate (%). **D** Immunoblot analysis of C3 and CC3. **E** Densitometric evaluation of C3, CC3, and β-tubulin. Mean ± SD (n = 6). #p < .05, vs. SC + saline group; *p < .05, Circ_26986 siRNA + LPS group vs. SC + LPS group

To determine the functions of mmu_Circ_26986 in BUMPT cell apoptosis after LPS treatment, mmu_Circ_26986 with siRNA was used. qRT-PCR analysis indicated that mmu_Circ_26986 siRNA suppressed its expression levels under LPS and basal conditions (Fig. 2A). Flow cytometry analysis demonstrated that mmu_Circ_26986 siRNA exaggerated LPS-stimulated BUMPT cell death (Fig. 2B&C), and this effect was verified by the immunoblotting results of cleaved-caspase3 (CC3) (Fig. 2D&E). These results suggest that mmu_Circ_26986 has an anti-apoptotic function during LPS treatment.

### Mmu_Circ_26986 overexpression ameliorates LPS-stimulated BUMPT cell apoptosis

**Fig. 3:**
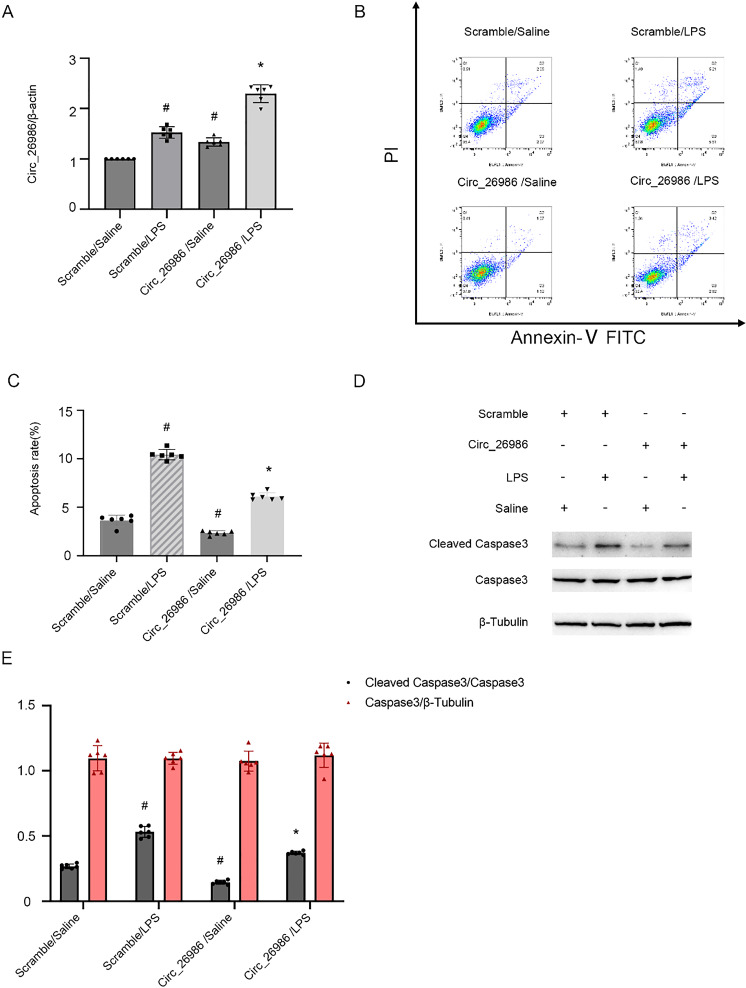
Circ_26986 overexpression attenuates LPS-stimulated apoptosis in BUMPT cells. BUMPT cell line was transfected with Circ_26986 plasmid or control, and subsequently exposed to LPS or no LPS for 24 h. **A** qRT-PCR detection of Circ_26986 levels. **B** Flow cytometry was utilized to evaluate BUMPT cell death. **C** Calculation of apoptotic rate (%). **D** Immunoblot assessment of C3. CC3 and β-tubulin. **E** Densitometric evaluation of immunoblot bands. Mean ± SD (n = 6). #p < .05, vs. SC + saline group; *p < .05, Circ_26986 + LPS group vs. SC + LPS group

To further support the above findings, the mmu_Circ_26986 plasmid was applied. qRT-PCR data indicated that overexpression of mmu_Circ_26986 upregulated its expression under LPS and basal conditions (Fig. 3A). Flow cytometry analysis showed that overexpression of mmu_Circ_26986 attenuated LPS-stimulated BUMPT cell death (Fig. 3B&C), and this effect was further verified by the immunoblotting results of CC3 (Fig. 3 D&E). These findings further confirm the data of mmu_Circ_26986 siRNA knockdown experiments.

### MiRNA-29b-1-5p is sponged by mmu_Circ_26986

**Fig. 4:**
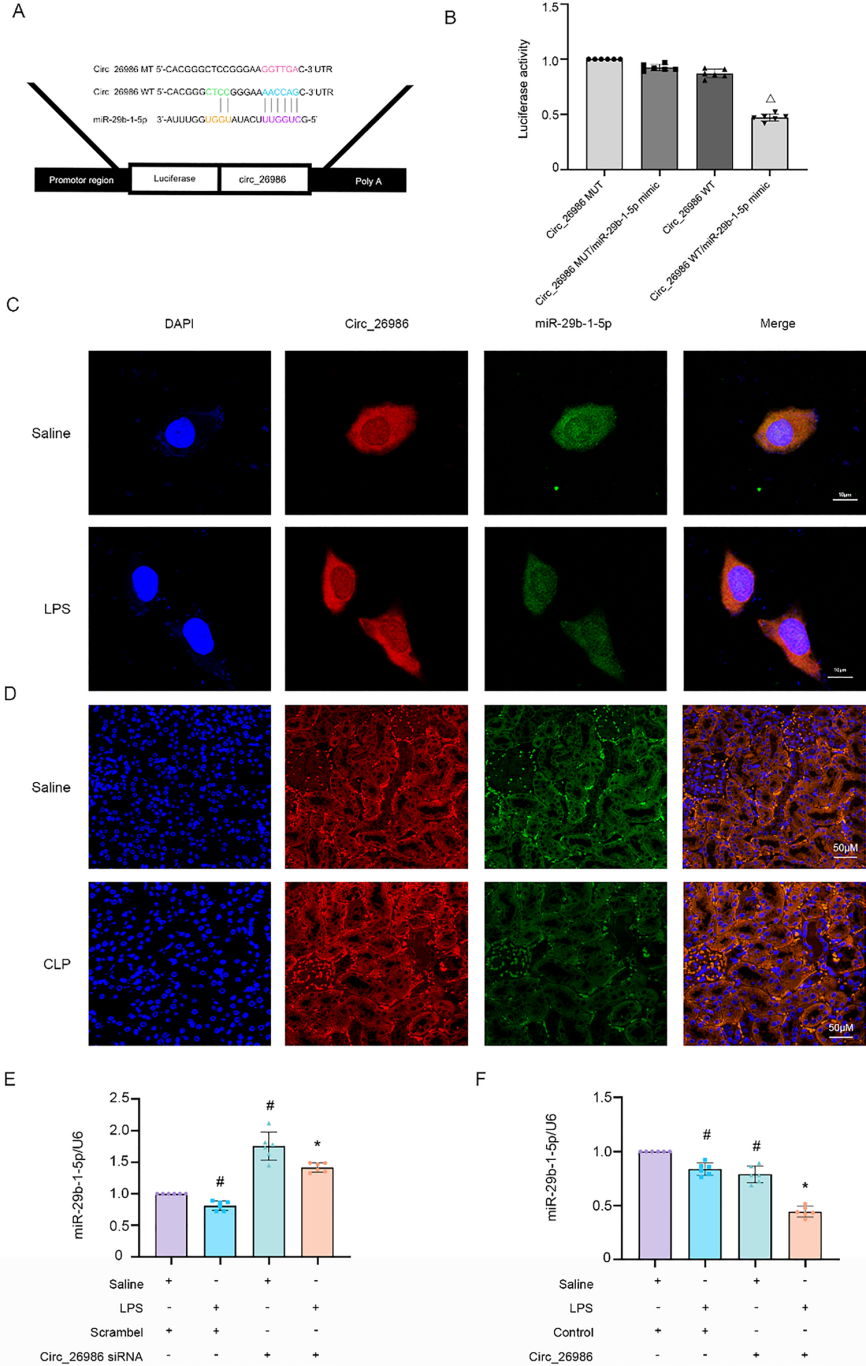
Circ_26986 inhibits miRNA-29b-1-5p activity and expression.** A** Complementary sequences of Circ_26986 and miRNA-29b-1-5p. **B** Determination of luciferase activity after Circ_26986-WT or Circ_26986-MUT co-transfection with miRNA-29b-1-5p mimic or SC. RNA-FISH for assessing the co-localization of Circ_26986 and miRNA-29b-1-5p in BUMPT cells (**C**) and kidney tissues (**D**) (Score bar: 50 µm) under basal or LPS conditions. **E**, **F** qRT-PCR analysis of miRNA-29b-1-5p levels following knockdown or overexpression of Circ_26986 and before and after LPS treatment. #p < .05, vs. SC + saline group; *p < .05, Circ_26986 siRNA or Circ_26986 plasmid + Saline group vs. SC or control + saline group; △p < .05, Circ_26986 WT/miRNA-29b-1-5p mimic vs. other groups

As mmu_Circ_26986 is overexpressed in the cytoplasm of BUMPT cells (Fig. 1J), we hypothesize that mmu_Circ_26986 may act as a miRNA sponge to modulate the expression of target genes. We found that Mmu_Circ_26986 contains a complementary binding site for miRNA-29b-1-5p via RegRNA v2.0 predication (Fig. 4A). Analysis of the luciferase reporter gene assay indicated that the miRNA-29b-1-5p mimic noticeably suppressed luciferase activity in the mmu_Circ_26986-wild-type (WT), while this effect was not observed in the mmu_Circ_26986-mutant (MUT) (Fig. 4B). Moreover, co-localization experiments indicated that mmu_Circ_26986 could interact with miRNA-29b-1-5p in the cytoplasm of basal and LPS-stimulated BUMPT cells as well as renal tubular cells of sham and CLP mice (Fig. 4C, D). Furthermore, qRT-PCR analysis demonstrated that LPS reduced the expression of miRNA-29b-1-5p, and this effect was enhanced and attenuated by the knockdown and overexpression of mmu_Circ_26986, respectively (Fig. 4E, F). Altogether, these data suggest that miRNA-29b-1-5p is a target of mmu_Circ_26986.

### MiRNA-29b-1-5p mimic exacerbates LPS-stimulated BUMPT cell apoptosis

**Fig. 5:**
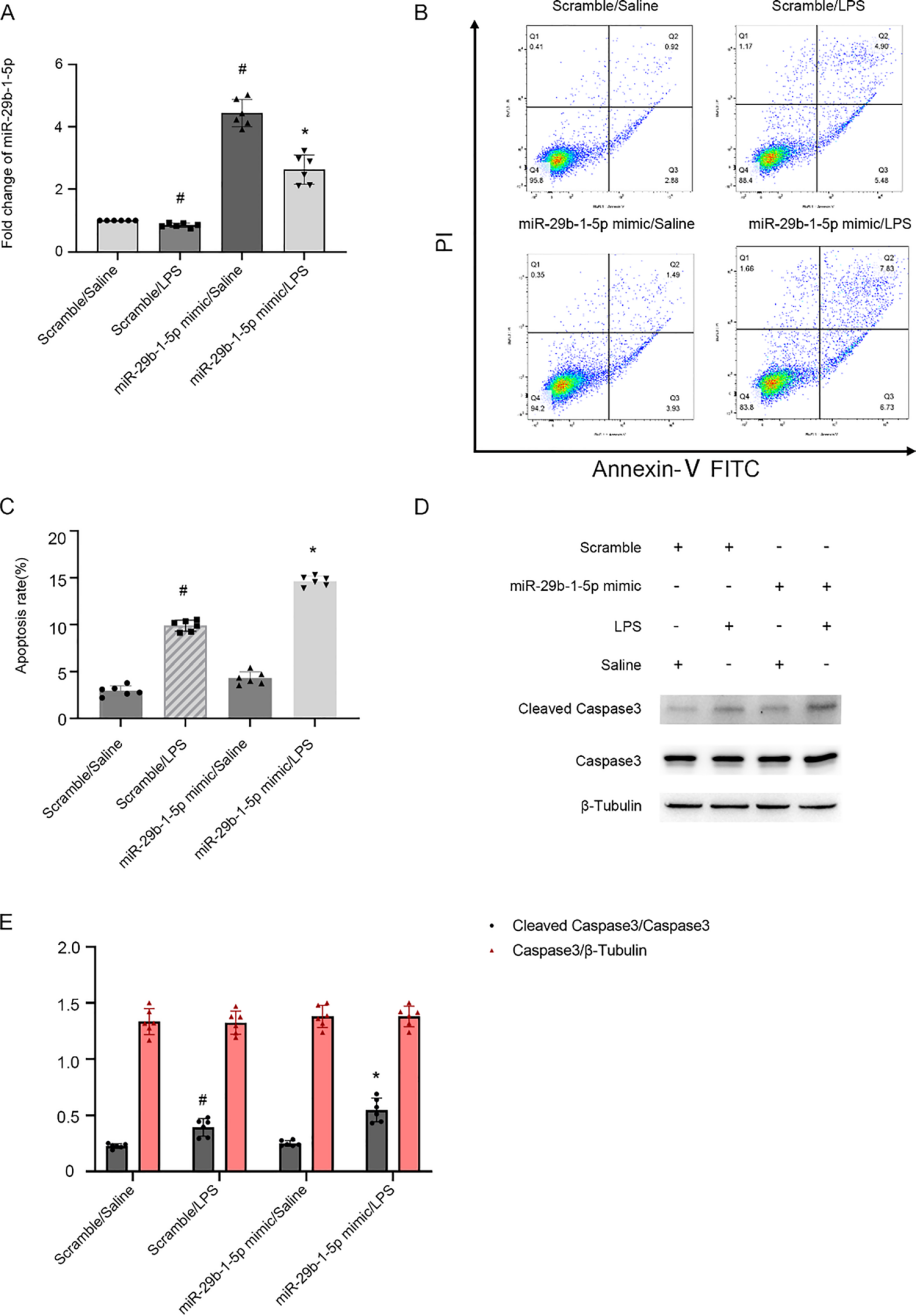
miRNA-29b-1-5p mimic exacerbate LPS-stimulated BUMPT cell apoptosis. Transfection of 100 nM miRNA-29b-1-5p mimic or SC prior to LPS damage in BUMPT cells. **A** Detection of miRNA-29b-1-5p expression by qRT-PCR. **B** Flow cytometry detection of BUMPT cell death. **C** Calculation of apoptosis rate (%). **D** Immunoblot assessment of C3, CC3 and β-tubulin. **E** Immunoblot band density analysis. Mean ± SD (n = 6). #p < .05, vs. SC + saline group; *p < .05, miRNA-29b-1-5p mimic with LPS, vs. SC + LPS group

Previous study has demonstrated the pro-apoptotic properties of miRNA-29b-1-5p in cardiomyocytes [17], but its role in renal cells apoptosis remains completely unknown. The qRT-PCR data indicated that miRNA-29b-1-5p mimic upregulated its expression under LPS and basal conditions (Fig. 5A). Flow cytometry analysis showed that miRNA-29b-1-5p mimic enhanced LPS-stimulated BUMPT cell death (Fig. 5B, C), and this effect was further validated by immunoblotting of CC3 (Fig. 5D, E). Thus, the findings suggest that miRNA-29b-1-5p is an apoptosis inducer.

### PAK7 is a target gene of miRNA-29b-1-5p

**Fig. 6:**
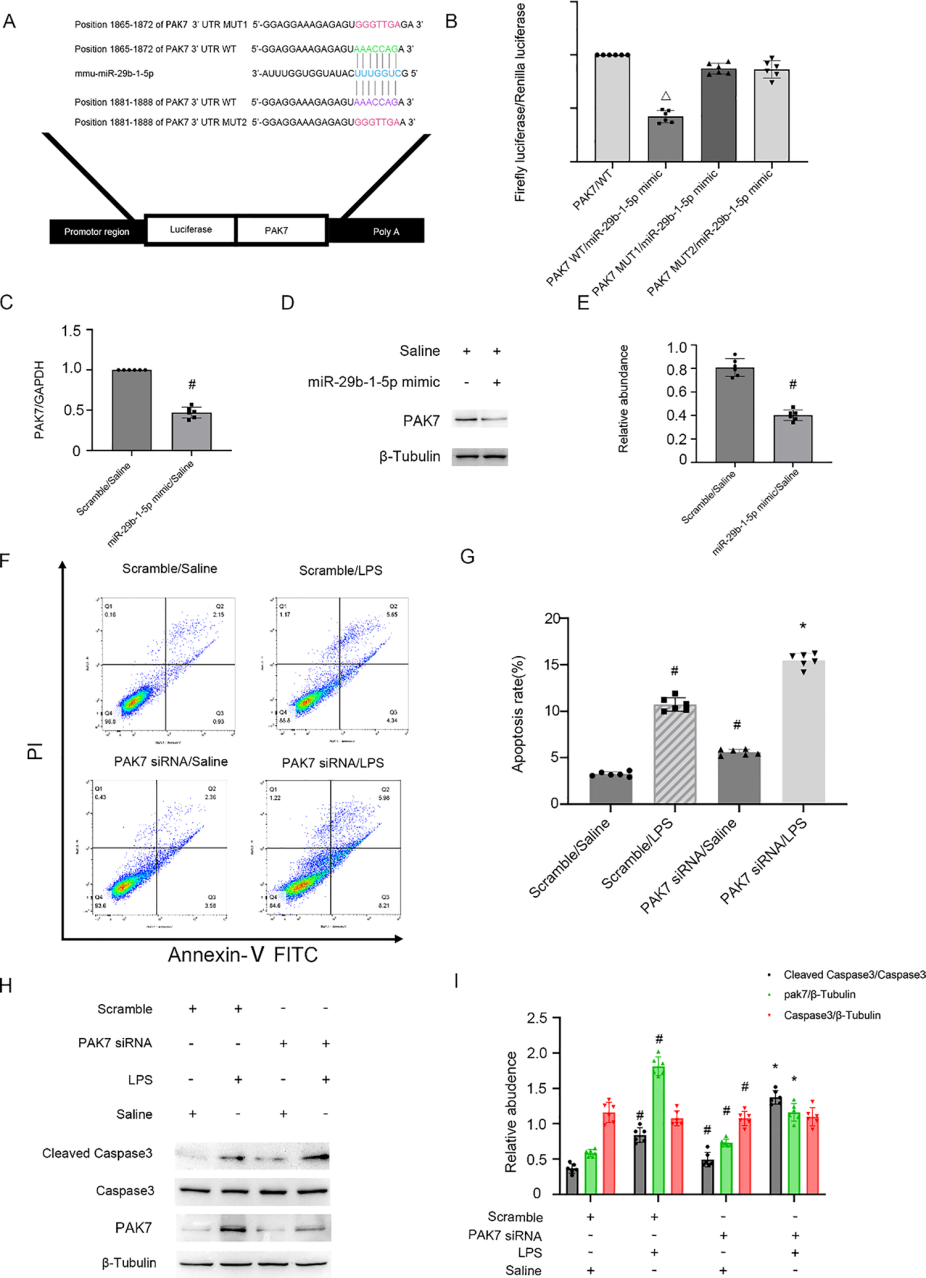
PAK7 is a direct target of miRNA-29b-1-5p. BUMPT cell line was transfected with miRNA-29b-1-5p mimic or PAK7 siRNA or SC, and then treated with/without (LPS 300 μg/mL) for 24 h. **A** miRDB database predicts that miRNA-29b-1-5p has a complementary binding site in the 3'-UTR of PAK7 mRNA. **B** miRNA-29b-1-5p was co-transfected with the 3’-UTR DLR vector of PAK7-1-MUT, PAK7-2-MUT or PAK7-WT, and the luciferase activities were then examined. **C** qRT-PCR detection of the mRNA expression of PAK7. **D** Immunoblot evaluation of PAK7 and β-tubulin levels. **E** Densitometric assessment of PAK7 and β-tubulin. **F**, **G** FCM analysis of BUMPT cell apoptosis. **H** Immunoblot analysis of C3, CC3 and PAK7. **I** Densitometric assessment of immunoblot bands. Mean ± SD (n = 6). #p < .05, vs. SC + Saline group; *p < .05, PAK7 siRNA + LPS group, vs. SC + LPS group. △p < .05, PAK7 WT/miRNA-29b-1-5p, vs. other groups

Several studies have reported that PAK7 exhibits anti-apoptotic functions in many cells [18,19]. However, its role in renal cells remains unclear. Interestingly, PAK7 was predicted to be a target gene of miRNA-29b-1-5p using miRDB database. The sequence analysis demonstrated that miRNA-29b-1-5p encompassed a complementary sequence of PAK7 (Fig. 6A). The luciferase reporters found that the miRNA-29b-1-5p mimic could suppress the luciferase activity of PAK7-WT but not PAK7-MUT (Fig. 6B). qRT-PCR and immunoblotting analysis revealed that miRNA-29b-1-5p mimic obviously decreased the mRNA/protein expression level of PAK7 (Fig. 6C–E). Flow cytometry analysis indicated that PAK7 siRNA remarkably increased LPS-stimulated BUMPT cell apoptosis (Fig. 6F, G), and this effect was verified by immunoblotting analysis of PAK7 downregulation and CC3 upregulation (Fig. 6H, I). This study confirms that PAK7 is a direct target gene of miRNA-29b-1-5p.

We further elucidated the mechanism underlying the anti-apoptotic action of PAK7 in BUMPT cell line. Immunoblot analysis showed that PAK7 siRNA reduced the expression of wnt and β-catenin, but promoted the expression of p- β-catenin (Supplement Fig. 1). These results demonstrate that PAK7 may play an anti-apoptotic role through the wnt/β-catenin pathway.

### The pro-apoptotic effect of mmu_Circ_26986 siRNA on BUMPT cell line upon LPS injury is reversed by miRNA-29b-1-5p inhibitor

**Fig. 7:**
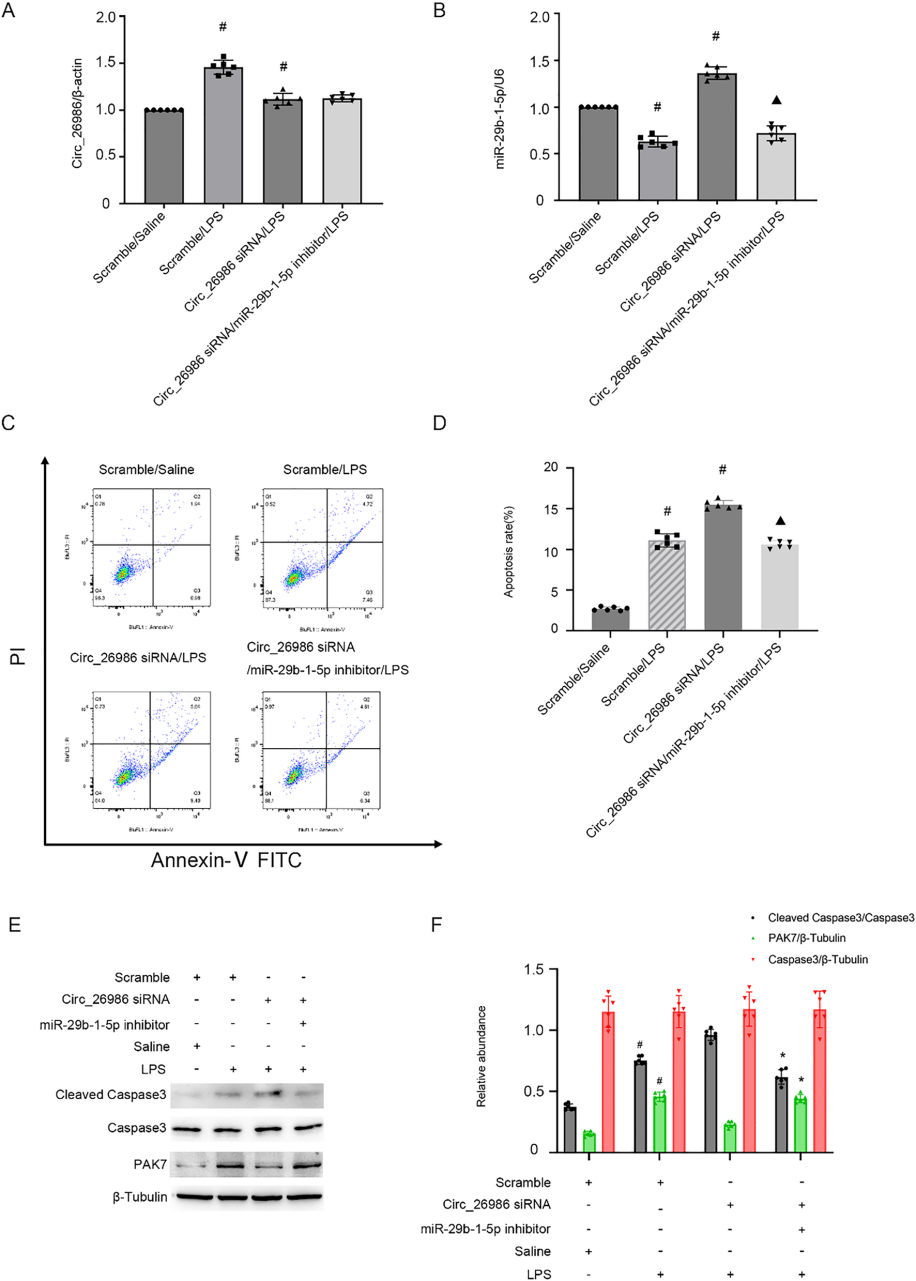
Downregulation of Circ_26986 enhances LPS-stimulated BUMPT cell apoptosis, while miRNA-29b-1-5p inhibitor reverses this process. BUMPT cell line was co-transfected with Circ_26986 (100 nM) and anti-miRNA-29b-1-5p or SC and then exposed to LPS for 24 h. **A**, **B** qRT-PCR assessment of Circ_26986 and miRNA-29b-1-5p levels. **C**, **D** Flow cytometry analysis of BUMPT cell apoptosis. **E** Immunoblot analysis of C3, CC3, PAK7 and β-tubulin. (F) Grey evaluation of immunoblot bands. Mean ± SD (n = 6). #p < .05, vs. SC + saline group; ▲p < .05, Circ_26986 siRNA + anti-miRNA-29b-1-5p + LPS group, vs. Circ_26986 siRNA + LPS group

We further confirmed whether miRNA-29b-1-5p mediated the anti-apoptotic effect of mmu_Circ_26986 during LPS treatment. The inhibition efficiencies of mmu_Circ_26986 siRNA and miRNA-29b-1-5p inhibitor were evaluated by qRT-PCR (Fig. 7A&B). Flow cytometry analysis showed that mmu_Circ_26986 knockdown enhanced LPS-stimulated apoptosis in renal cells, which could be reversed by the miRNA-29b-1-5p inhibitor (Fig. 7C&D). Immunoblotting results of the changes in cleaved-caspase-3 and PAK7 further supported the flow cytometry data (Fig. 7E&F). The findings further verified that mmu_Circ_26986 suppressed LPS-stimulated renal cell apoptosis via the miRNA-29b-1-5p/PAK7 axis.

### Overexpression of mmu_Circ_26986 attenuates SA-AKI in mice by regulating the miRNA-29b-1-5p/Pak7 axis

**Fig. 8:**
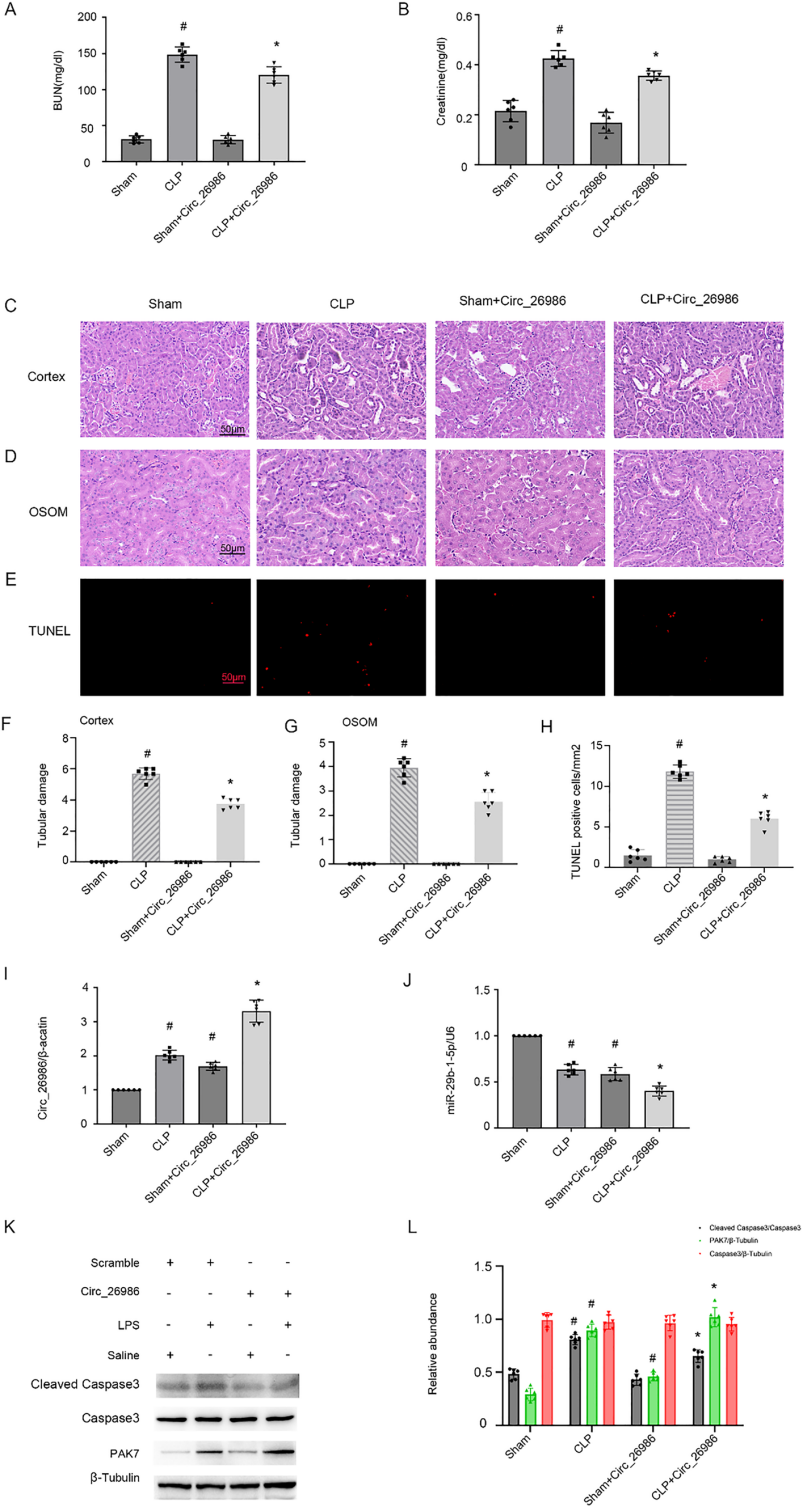
LPS-stimulated AKI in male C57BL/6 mice can be suppressed by Circ_26986 overexpression. C57BL/6 mice were given Circ_26986 plasmid via tail vein for 12 h, and subsequently received CLP for 18 h or a sham-operated group as a control. Determination of serum BUN (**A**) and creatinine (**B**) concentrations. **C** Staining of renal cortex with hematoxylin and eosin. **D** H&E staining of the renal medulla. **E** TUNEL staining was also performed on the kidneys. Score bar: 50 µm. Renal cortical (**F**) and OSOM (**G**) tubular injury scores. **H** TUNEL-positive cell count. **G**, **H** qRT-PCR assessment of Circ_26986 and miRNA-29b-1-5p. **I** Immunoblot evaluation of C3, CC3, and PAK7. **J** Densitometric evaluation of immunoblot bands. Mean ± SD (n = 6). #p < .05, control + CLP or Circ_26986 plasmid group, vs. saline group; *p < .05, Circ_26986 plasmid + CLP group, vs. control + CLP group

To better evaluate the functions of mmu_Circ_26986 in SA-AKI, C57BL/6 J mice were exposed to mmu_Circ_26986 overexpression plasmid for 12 h via tail vein and then subjected to CLP treatment. Overexpression of mmu_Circ_26986 markedly suppressed CLP-induced elevation of serum creatinine and BUN (Fig. 8A, B). Consistently, H&E and TUNEL staining results demonstrated that overexpression of mmu_Circ_26986 attenuated CLP-induced renal tubule damage of outer medullary cortex and outer stripe (OSOM) (Fig. 8C, F, D, G) and renal cell apoptosis (Fig. 8E, H). The overexpression of mmu_Circ_26986 markedly increased mmu_Circ_26986 mRNA expression, while suppressed miRNA-29b-1-5p expression (Fig. 8I, J). Immunoblotting results showed that overexpression of mmu_Circ_26986 suppressed CLP-stimulated expression of PAK7 and cleaved-caspase-3 (Fig. 8K, L). Collectively, our study reveals that overexpression of mmu_Circ_26986 prevents the progression of SA-AKI mice via the miRNA-29b-1-5p/PAK7 axis.

### Overexpression of hsa_Circ_0072463 inhibits LPS-stimulated HK-2 cell apoptosis

**Fig. 9:**
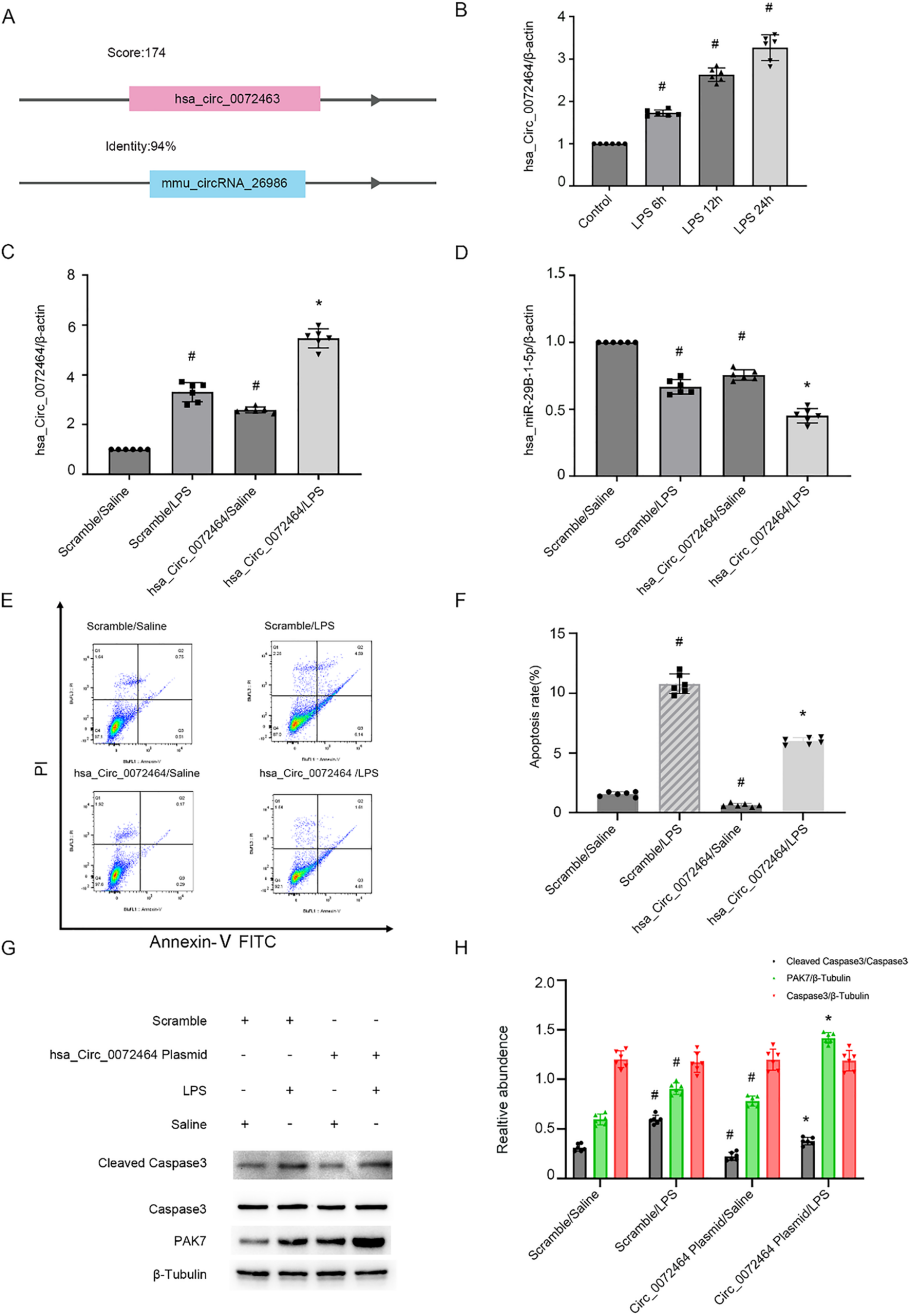
Overexpression of hsa_Circ_0072463 attenuates LPS-stimulated HK-2 cell apoptosis.** A** Sequence comparison of mmu_Circ_26986 and hsa_Circ_0072463. Sequence conservativeness was examined using the blast function of CircBase webtool (http://circrna.org/cgi-bin/webBlat). According to the blast result, when the homology > 80%, the higher the score, the higher the homology of hsa_Circ_RNA is considered. HK-2 cells were exposed to LPS (50 μg/mL) for 6, 12 or 24 h. **B** qRT-PCR assessment of hsa_Circ_0072463 levels in cells. HK-2 cell line was transfected with hsa_Circ_0072463 plasmid and then were subjected to LPS 24 h. qRT-PCR was utilized for examining hsa_Circ_0072463 (**C**) and hsa-miRNA-29b-1-5p (**D**) levels. **E** Flow cytometry evaluation of HK-2 cell apoptosis. **F** Calculation of apoptosis rate. **G** Immunoblot assessment of the expression of C3, CC3, and PAK7 in HK-2 cells. **H** Grayscale evaluation of the immunoblot bands of C3, CC3, and PAK7 in HK-2 cells. Mean ± SD (n = 6). #p < .05 vs. SC + saline group. *p < .05 vs. SC + I/R group

To further explore whether mmu_Circ_26986 has a homology with human CircRNA, the Circbase database was used. We found that mmu_Circ_26986 with homology hsa_Circ_0072463 was the most likely target (Fig. 9A). We further investigated the expression of hsa_Circ_0072463 upon LPS exposure. The qRT-PCR results demonstrated that hsa_Circ_0072463 was overexpressed at 6 h and peaked at 24 h (Fig. 9B). To validate the function of hsa_Circ_0072463, hsa_Circ_0072463 plasmid was transfected into HK-2 cell line and subsequently exposed to LPS. The qRT-PCR data revealed that overexpression of hsa_Circ_0072463 increased its expression but reduced miRNA-29b-1-5p expression under the basal and LPS conditions (Fig. 9C, D). The flow cytometry analysis results showed that hsa_Circ_0072463 overexpression noticeably ameliorated LPS-stimulated HK-2 cell death (Fig. 9E, F), which was validated by the immunoblot analysis of CC3 protein downregulation and PAX7 upregulation (Fig. 9G, H). These findings imply that hsa_Circ_0072463 shares the same function and mechanism with mmu_Circ_26986 during LPS stimulation.

### Homologous hsa_Circ_0072463 of mmu_Circ_26986 is a biomarker of SA-AKI

**Fig. 10:**
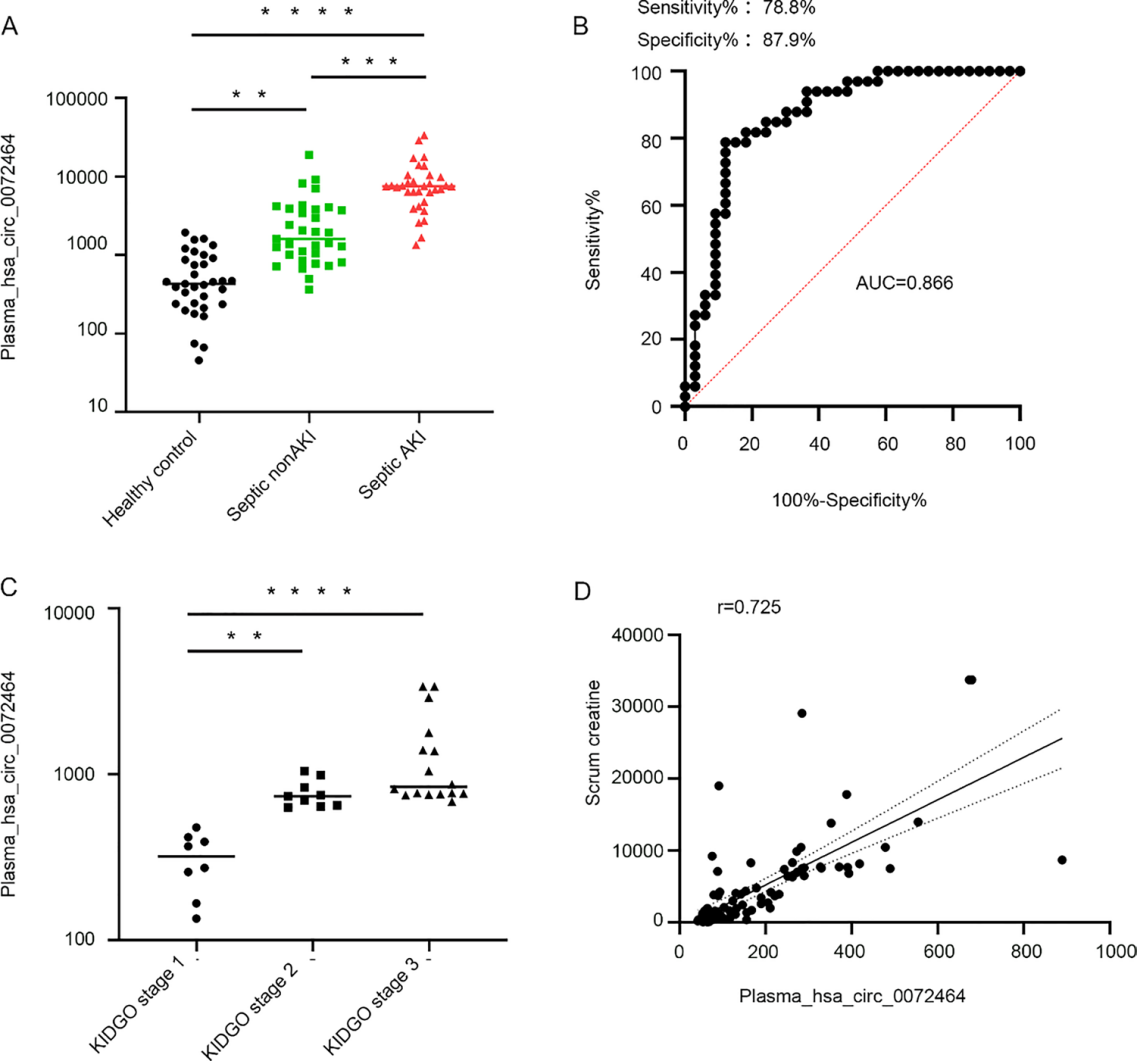
Hsa_Circ_0072463 as a diagnostic marker for AKI**. A** Absolute RT-PCR analysis of plasma Circ_0072463. **B** AUC-ROC curves for the hsa_Circ_0072463 diagnosis of SA-AKI vs. SA-non-AKI. **C** Correlation coefficients between hsa_Circ_0072463 and creatinine. **D** The levels of hsa_Circ_0072463 in different stages of SA-AKI. *p < .05, **p < .01, ***p < .001, ****p < .0001

To evaluate whether plasma hsa_Circ_0072463 could act as a diagnostic marker in patients with SA-AKI, we collected the plasma specimens from SA-AKI patients (n = 33), non-AKI patients (n = 33), and age-matched healthy subjects (n = 33), and the expression of hsa_Circ_0072463 was then analyzed. Absolute RT-PCR evaluation indicated that the expression of hsa_Circ_0072463 was induced in septic non-AKI compared with controls, and its expression was higher in sepsis patients than in septic non-AKI patients (Fig. 10A). The clinical features of SA-AKI and non-SA-AKI patients as well as age-matched healthy subjects (n = 99) are shown in Supplement Table 1. We further assessed the diagnostic potential of hsa_Circ_0072463 by generating a subject work characteristics (ROC) curve. The sensitivity, specificity and AUC of plasma hsa_Circ_0072463 were 78.8%, 87.9% and 0.866 (95% confidence interval 0.775–0.957), respectively (Fig. 10B). Interestingly, the expression level of plasma hsa_Circ_0072463 in KIDGO stage2 was higher compared to KIDGO stage1. However, its expression level in KIDGO stage3 was not significantly increased compared to KIDGO stage2 (Fig. 10C). Spearman's correlation coefficient showed the positive correlation between plasma hsa_Circ_0072463 levels and serum creatinine (Fig. 10D). In summary, hsa_Circ_0072463 may function as an early diagnostic marker for SA-AKI.

## Discussion

The mounting evidence suggests that CircRNAs are responsible for the development of SA-SKI [15,20–22]. In our research, we demonstrated for the first time that mmu_Circ_26986 has a protective effect against LPS-stimulated BUMPT cell apoptosis (Figs. 2, 3). Mechanistically, Mmu_Circ_26986 acts as a ceRNA, targeting miRNA-29b-1-5p to enhance the expression of the antiapoptotic protein PAK7. Overexpression of mmu_Circ_26986 effectively hinders the progression of CLP-induced SA-AKI (Fig. 8). Furthermore, we identified hsa_Circ_0072463, the homologous of mmu_Circ_26986, as an early diagnosis biomarker for SA-AKI (Fig. 10).

Previous reports have highlighted the significant role of CircRNAs in mediating in renal tubular cell apoptosis during sepsis injury [23,24]. Various CirRNAs such as Circ_35953, Circ_HIPK3, Circ_0114427, CIRC_0114428, Circ_0001714, circ_0020339, Circ_RASGEF1B, Circ_0114428, circ-FANCA, CircNRIP1, circ0001818, and Circ_0001806 [16,23,25–34] have been implicated in LPS-induced renal tubular cell apoptosis. Conversely, others such as CircITCH, CircVMA21, CIRC_0008882, and Circ_0091702 [35–38] exhibit an opposing role. In our present study, we highlight the antiapoptotic role of mmu_Circ_26986, substantiated by both overexpression and knockdown experiments (Figs. 2, 3). Our data strongly support the notion that mmu_Circ_26986 possesses anti-apoptotic potential.

CircRNAs are known to act as sponges for microRNAs in the cytoplasm, thereby increasing the expression of downstream genes [39–41]. The FISH analysis showed that mmu_Circ_26986 predominantly localized in the cytoplasm (Fig. 1). Next, we predicated and verified that miRNA-29b-1-5p could be directly targeted mmu_Circ_26986 (Fig. 4). However, the function of miRNA-29b-1-5p in apoptosis has been a subject of controversy. While one study reported its suppression of LPS-induced apoptosis in MH-S cells [41], another indicated its involvement in mediating apoptosis in hydrogen peroxide-treated cells by targeting Bcl2 [42]. Our data align with the latter, demonstrating that miRNA-29b-1-5p mediates renal tubular cells apoptosis via targeting p21-activated kinase 7 (PAK7) (Fig. 6). Previous studies have reported that PAK7 exerts an antiapoptotic function in multiple tumor cell lines [43–45]. Similarly, our findings show that PAK7 knockdown enhances LPS-induced apoptosis in BUMPT cells through the Wnt/β-catenin pathway (Fig. 6). The mmu_Circ_26986/miRNA-29b-1-5p/PAX7 axis was further validated through in vitro recovery experiments and in vivo overexpression of mmu_Circ_26986 (Fig. 8). Collectively, our study indicates that the mmu_Circ_26986/miRNA-29b-1-5p/PAK7 axis plays a pivotal role in the development of SA-AKI.

A recent study highlighted circ_0020339 as an early diagnostic marker for SA-AKI, but the methodology lacked sensitivity and specificity calculations [16]. In our research, we identified, for the first time, a homologue of mmu_Circ_26986, hsa_Circ_0072463, which mediates LPS-stimulated HK-2 cell apoptosis through the regulation of the miRNA-29b-1-5p/PAK7 axis. Furthermore, we validated hsa_Circ_0072463 as an early diagnostic marker for SA-AKI, as supported by its higher sensitivity of 78.79%, higher specificity of 87.88%, and a stronger correlation with serum creatinine (a conventional diagnostic marker of kidney injury) (Fig. 10). However, it is important to note that our study is limited by the sample size, and future research should expand the cohort to further explore the diagnostic potential of hsa_Circ_0072463 for SA-AKI.

**Fig. 11:**
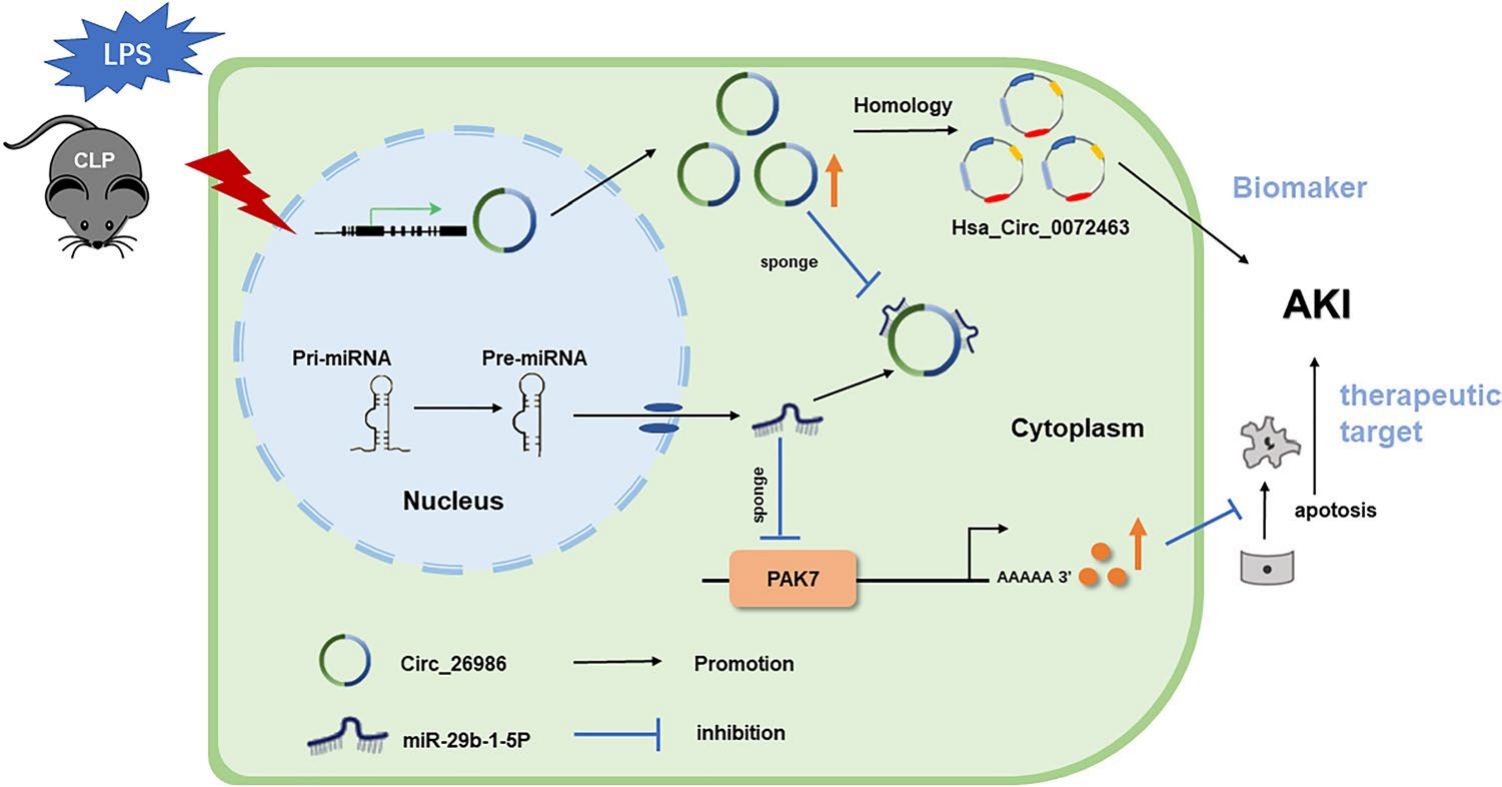
Modelling and mechanism of mmu_Circ_26986/hsa_Circ_0072463 in septic AKI. In sepsis AKI, the expression of mmu_Circ_26986 is increased. mmu_Circ_26986 competitively acts on miRNA-29b-1-5p, increases PAK7 expression, and exhibits anti-apoptotic effects on cell and animal models. Homologous hsa_Circ_0072463 is the most likely target of mmu_Circ_26986. Hsa_Circ_0072463 may serve as an early diagnostic marker and a new therapeutic target for septic AKI

In conclusion, our data reveal that mmu_Circ_26986/hsa_Circ_0072463 exerts an anti-apoptotic function by targeting the miRNA-29b-1-5p/PAK7 axis. These CircRNAs hold promise as therapeutic targets for SA-AKI, with hsa_Circ_0072463 emerging as a reliable biomarker for the condition (see Fig. 11).

### Supplementary Information

Below is the link to the electronic supplementary material.

Supplementary file1 (DOCX 3066 KB)

## Data Availability

Primary data will be available from the authors upon request.

## Abbreviations

AKI: Acute kidney injury
AUC: The area under the ROC curve
BUMPT: Boston University mouse proximal tubule
BUN: Blood Urea Nitrogen
CC3: Cleaved caspase-3
CLP: Cecum ligation and puncture
CircRNA: Circular RNA
ceRNA: Competing endogenouse RNA
HK-2: Human renal tubular epithelial cells
LPS: Lipopolysaccharide
PAK7: P21 (RAC1) activated kinase 7
RT-qPCR: Real-time fluorescence quantitative PCR
ROC: Receiver operating characteristic
SA-AKI: Sepsis AKI

## Availability of data and materials

All data supporting the findings of this study are available within the paper and its Supplementary Information.
