# Supplementary material for: Discovery and verification of mmu_Circ_26986/hsa_Circ_0072463 as a potential biomarker and intervention target for sepsis-associated acute kidney injury
Source: Cell Mol Life Sci. 2024 Mar 28;81(1):154. doi: 10.1007/s00018-023-05079-x (PMC10973092; doi:10.1007/s00018-023-05079-x)
Supplement: Supplementary file 1 — Supplementary file1 (DOCX 3066 KB) [file 18_2023_5079_MOESM1_ESM.docx]

**Supplemental Materials for**

**Discovery and verification of** **mmu_Circ_26986/hsa_Circ_0072463 as a potential biomarker and** **intervention target for sepsis-associated acute kidney**

Xujun Peng^1,2,3^, Huiling Li^3^, Wenbo Zhang^5^, Dongshan Zhang^1,2,4^*

^1^ Department of Emergency Medicine, ^2^ Emergency Medicine and Difficult Diseases Institute, ^3^ Department of Ophthalmology, ^4^Department of Nephrology, Second Xiangya Hospital, Central South University, Changsha, Hunan, People’s Republic of China. ^5^Boya College of Macau University of Science and Technology.

***Running Title:*** The role of **mmu_Circ_26986/hsa_Circ_0072463** in septic AKI

* ***Correspondence address:***

Dongshan Zhang

^1^Department of Emergency Medicine, ^2^Emergency Medicine and Difficult Diseases Institute, ^5^Department of Nephrology, Second Xiangya Hospital, Central South University, Changsha, Hunan 410011, People’s Republic of China. Tel +86 138 7589 9625. Email: [dongshanzhang@csu.edu.cn](mailto:dongshanzhang@csu.edu.cn).

Key words –mmu_Circ_26986; hsa_Circ_0072463; biomaker；AKI；SA-AKI；apoptosis；LPS；CLP; miRNA-29b-1-5p; PAK7;

[Cellular and Molecular Life Sciences](https://www.springer.com/journal/18)


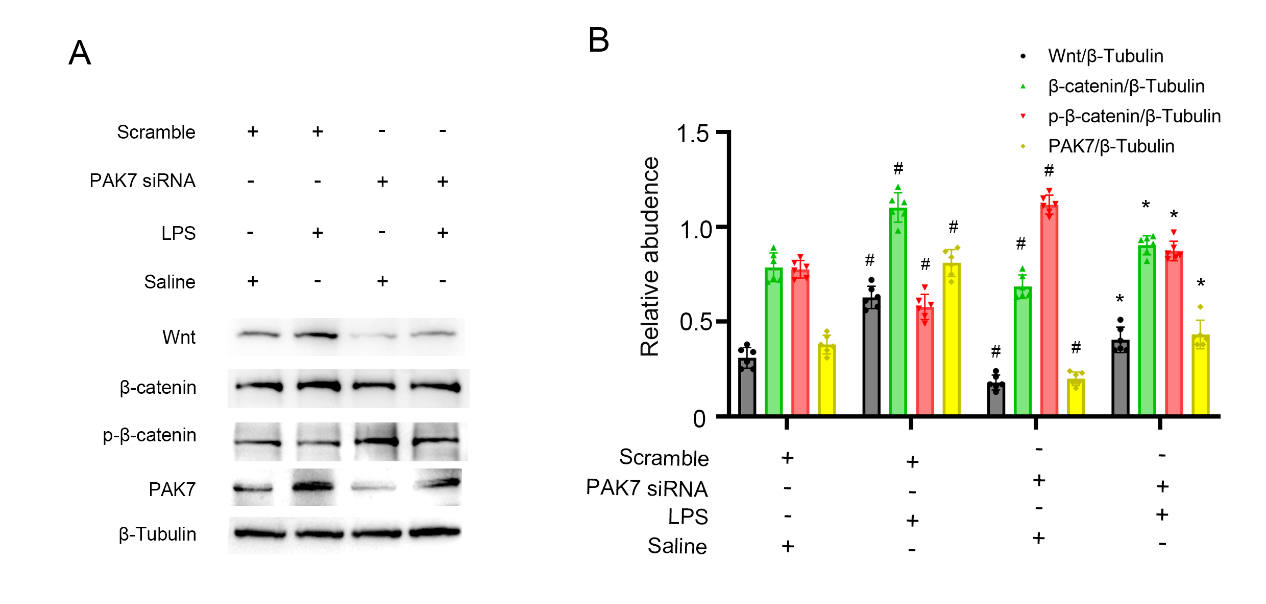


**Supplemental Fig1. PAK7 activates the Wnt/β-catenin signalling pathway in SA-AKI.**

BUMPT cell line was transfected with PAK7 siRNA or SC, and then treated with/without (LPS 300 μg/mL) for 24 hours. (A) Immunoblot evaluation of PAK7,Wnt, β-catenin，p-β-catenin and β-tubulin levels. (B) Grey evaluation of immunoblot bands. Mean±SD (n=6). #p<.05, vs. SC + Saline group; *p<.05, PAK7 siRNA + LPS group, vs. SC + LPS group.

**Supplemental S1. General patient characteristics**

**
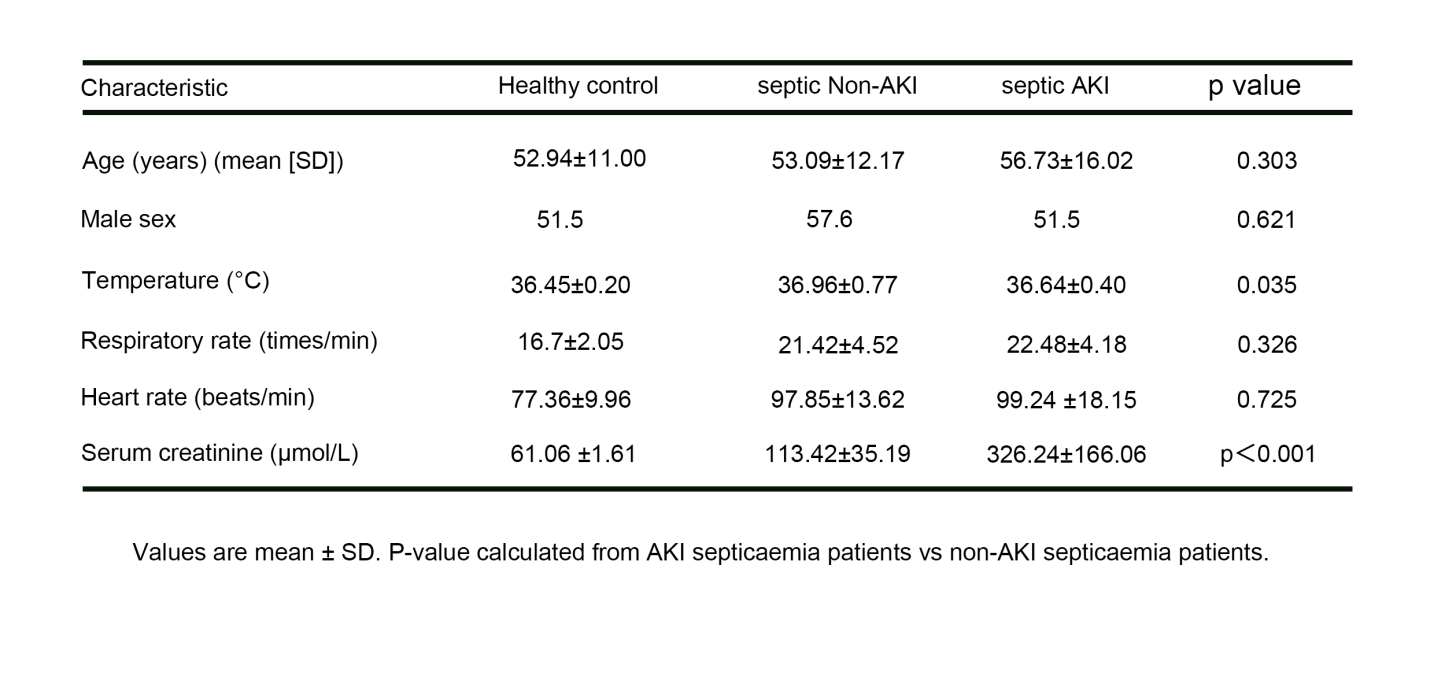
**
